# The Frustration-induced Ferroelectricity of a Manganite Tricolor Superlattice with Artificially Broken Symmetry

**DOI:** 10.1038/s41598-017-06640-y

**Published:** 2017-07-24

**Authors:** Huanyu Pei, Shujin Guo, Lixia Ren, Changle Chen, Bingcheng Luo, Xianglei Dong, Kexin Jin, Ren Ren, Hafiz Muhammad Zeeshan

**Affiliations:** 1 Shaanxi Key Laboratory of Condensed Matter Structures and Properties, Northwestern Polytechnical University, Xi’an, 710072 China; 2 Department of Physics, Xi’an Jiaotong University, Xi’an, 710072 China

## Abstract

In this paper, [(La_0.9_Sr_0.1_MnO_3_)_n_/(Pa_0.9_Ca_0.1_MnO_3_)_n_/(La_0.9_Sb_0.1_MnO_3_)_n_]_m_ superlattices films have been deposited on (001) Nb:SrTiO_3_ substrates by a laser molecular-beam epitaxy technology. Expected ferroelectricity arise at well-defined tricolor superlattice at low temperature, composed of transition metal manganite, which is absent in the single-phase compounds. Furthermore, the ferroelectric properties of the superlattices are enhanced by increasing the periodicity m, which may be attributed to the accumulation of the polarization induced by the frustration. As for the magnetic hysteresis loop characteristics of the multilayer structures, the saturation magnetization and magnetic coercivity of films present definitely a strong periodic dependence. It also indicates that the frustration may exist in the tricolor superlattice. Our results further verify the previous theoretical research of generating multiferroics experimentally paving a way for designing or developing the novel magnetoelectric devices based on manganite ferromagnets.

## Introduction

Single-phase multiferroics, which feature simultaneously more than one spontaneous primary ferroic order parameter where the order parameters can be coupled, exhibit unusual physical cross-correlation effects as widely potential applications, thus it has become one of the hottest disciplines of condensed matter physics and materials science in recent years^1, 2^. From the point of view of symmetry consideration, ferroelectricity and magnetism need different broken inversion symmetries in micro-electronic states, which leads to the incompatibility between ferroelectricity and magnetism in single-phase materials. Among all of the 233 Shubnikov magnetic point groups, only 13 point groups allow the simultaneous appearance of spontaneous polarization and magnetization^3^. This restriction in the crystallographic symmetry results in the fact that single-phase multiferroics are rare in nature^4^. So far, scientists have discovered a few single-phase multiferroics, such as YMnO_3_, TbMnO_3_, LuFe_2_O_4_ and BiFeO_3_^5–10^. Unfortunately, there are no significant breakthroughs of the research about single-phase multiferroics, which encounter a bottleneck. So far, no reports have appeared on the practical application of multiferroics, illustrating the fact that there is much work still to be done to make the multiferroics strategy a practical reality^11^. Therefore, approaches different from simple system considerations are essentially required. Exploring the microscopic mechanism of ferroelectricity and ferromagneticity is a hot topic of improving the material properties and applications. In view of this, several multiferroic physical mechanisms for different materials have been proposed, including the lone-pair mechanism^12^, geometric ferroelectricity in hexagonal manganites^13^, spiral spin-order-induced multiferroicity^14^, off-centre shifts in geometry, frustrated magnets with competing interactions between spins and complex magnetic orders^15^, and so on. A multiferroic system requires the simultaneous breaking of the spatial inversion and time-reversal symmetries^16–18^. So regulating and strengthening the multiferroics by adopting the low-dimensional films with structural asymmetry become viable candidates. Tokura Y believed that the tricolor superlattices can be viewed as tailormade multiferroics^19^. Due to the broken space inversion symmetry in asymmetric SLs of tri-layers, the toroidal moments are not cancelled but rather accumulated, giving rise to the ferrotoridic tailor-made compound in this structure contrasted to bicolor SLs.

Doped manganite with perovskite structure, Re_1-x_Ae_x_MnO_3_ (Re: rear-earth ion; Ae: doping ion), is known as a compound for the strong correlation^20^. The pure LaMnO_3_ and PrMnO_3_ are A type antiferromagnet. The magnetic structure is generally associated with the Mn-O-Mn bond angle in pure ReMnO_3_,. A systematic change in crystal structure by changing Re ion is characterized by the Mn-O-Mn bond angle; a deviation of the bond angle from 180° becomes steep by changing Re from La to Ho in the periodic table^20^. Moreover, doping Ae ions may bring the charge transfer. This transfer of charge from the Mn sites to the Mn–O–Mn bonds can drives the bond-length shortening^21^. Many doped compounds are ferromagnets. For example, below T = 70 K the magnetic structure of Pr_0.9_Ca_0.1_MnO_3_ is non-collinear with manganese moments lying in the xy plane^22^. In view of this, we report on the growth and properties of high-quality tricolor superlattices (SLs) composed of transition-metal oxides to create multiferroics in this paper. SLs are grown with an asymmetric A-B-C-A-B-C… stacking order consisting of layers of hole-doped Pr_0.9_Ca_0.1_MnO_3_ (PCMO, A), La_0.9_Sr_0.1_MnO_3_ (LSMO, B) and electron-doped La_0.9_Sb_0.1_MnO_3_ (LSbMO, C). As mentioned above, tricolor superlattice composed of different materials, can result in the breaking of the spatial inversion symmetric ultimately forming a frustrated system. Furthermore, the characterization on ferroelectricity and magnetism at different temperatures has been made along with the investigation of their dependency on the thickness of sublayers. The tricolor superlattice has already been proposed more than 15 years, and some similar oxide compound superlattices have been demonstrated^23–26^. The idea used in this research is different from previous work. First, all three compounds are ferromagnetics. Second, they are non-ferroelectrics^27–29^. The aim of this research is the emergence of the ferroelectricity from the frustrated system, instead of the intrinsic nature of the materials. The related experimental work are reported rarely.

## Results and Discussion

**Figure 1:**
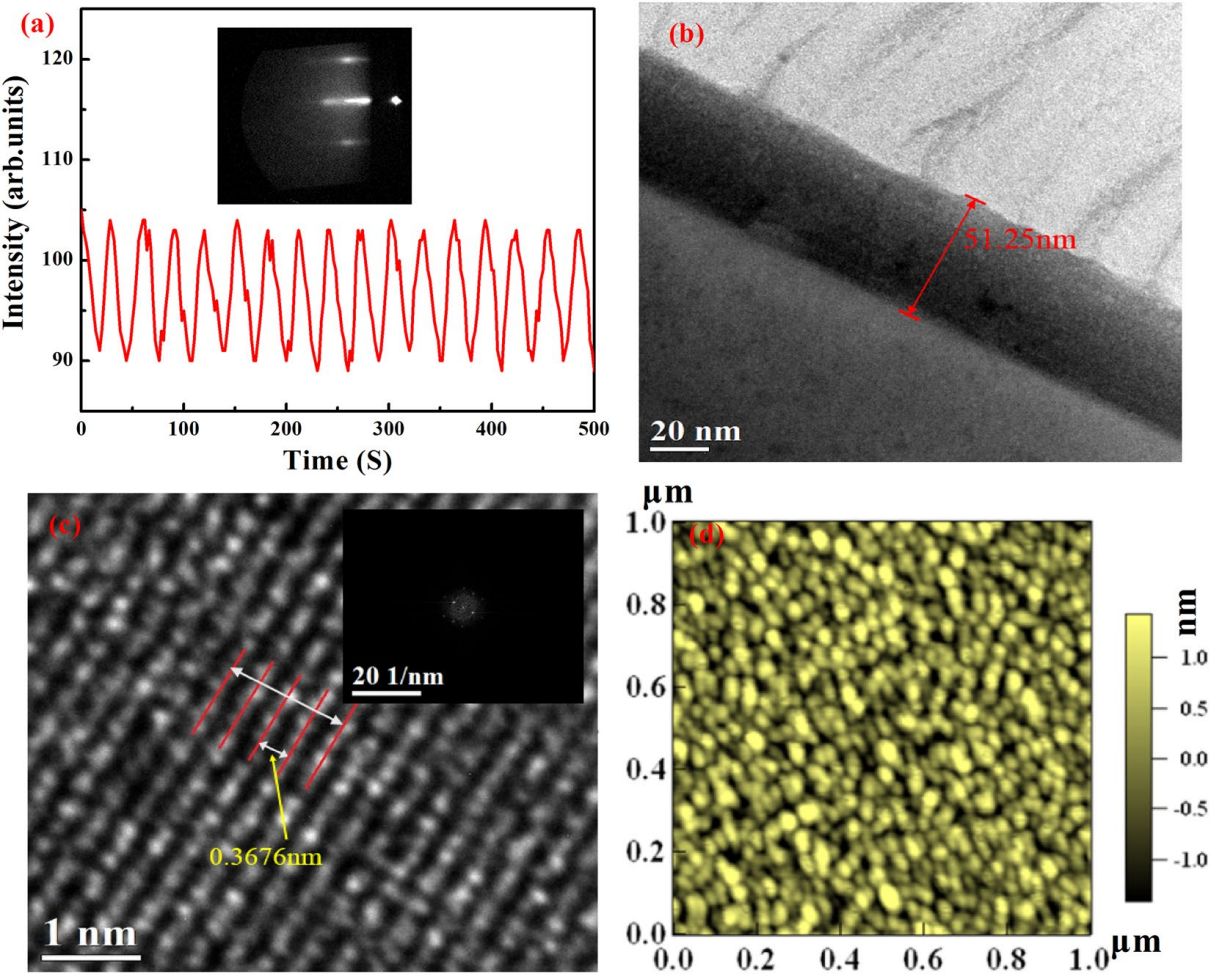
Details about RHEED pattern and the sample structure. (**a**) RHEED patten and intensity oscillations during deposition. Cross-sectional morphologies (**b**), macroscopic structure and FFT patten (**c**) and surface (**d**) of the SL film.

The [(LSMO)_n_/(PCMO)_n_/(LSbMO)_n_]_m_ SLs are deposited on the Nb:SrTiO_3_ (NSTO) single crystal substrate using a laser molecular-beam epitaxy (L-MBE) deposition method. Labeling of superlattice structure is carried in terms of (*n, m*), where *n* is the thickness of each sublayers and *m* indicates the periodicity of alternating cyclically LSMO, PCMO and LSbMO sublayers. The reflection high-energy electron diffraction (RHEED) specular beam intensity oscillation as a function of growth time is shown in Fig. 1(a). The observed *in situ* RHEED oscillation reflects a clean layer by layer u.c. growth. The inset of Fig. 1(a) shows the RHEED pattern observed for SL film. Figure 1(b) is the cross-sectional transmission electron microscopy (TEM) observation. Thickness of SL film is determined to be about 51.25 nm. Figure 1(c) is a partially enlarged image with 1 nm × 1 nm dimensions, and the size of a unit-cell is about 0.3676 nm. The inset is the fast Fourier transform (FFT) of Fig. 1(c) which indicates that the film has a good orientation. Otherwise, Fig. 1(d) acquired by atomic force microscope (AFM) demonstrates that the film has an atomistic precision, and the corresponding root-mean-square roughness is 0.56 nm. In one word, the film is epitaxially grown with high quality.

**Figure 2:**
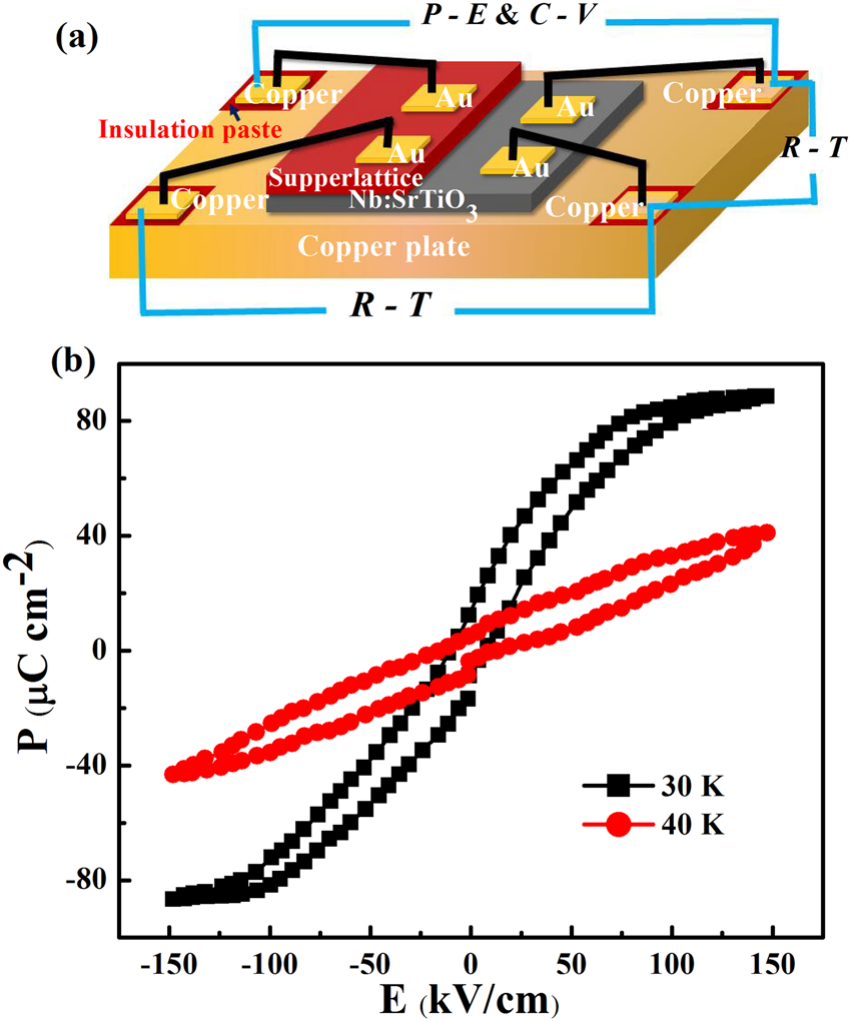
(**a**) Schematic view of the four electrodes fabricated on SLs. (**b**) Polarization as function of electric field (*P-E*) in the multilayer film measured at 30 K and 40 K at the frequency of 2 kHz.

Then, we measure the resistance of SL film which exceeds the measure limit ~10^12^ Ω of instrument. The film has a good insulation performance. Figure 2(a) is a configuration diagram of film and electrode. The sample is cooled and warmed at a rate of 3 K per minute, respectively. The obtained data reveal that the cooling or warming process has no influences on the results. As a representative, Fig. 2(b) shows the polarization versus electric field (*P-E*) hysteresis loops measured with warming at 30 K and 40 K. The remnant polarization (*Pr*) and the saturation polarization (*Ps*) at 30 K are 18.9 μC cm^−2^ and 88.7 μC cm^−2^, respectively. The *P-E* loops above 40 K and below 30 K can be found as Supplementary Fig. S1. The *P-E* loop of film presents ferroelectric behavior below 30 K, which is distinguished from the suppression of polarization above 40 K. The three kinds of materials constituted the film are strongly correlated, and possess the coupling of lattice, charge, orbital, spin, and so on. Each of the interactions in the competition between them tends to favor its own characteristic spatial correlations^30, 31^. Especially in the multilayer structure of system, the existence of the interface or surface of SLs leads to lattice distortions and charge reconstruction, which can result in the breaking of the spatial inversion symmetric ultimately forming a frustrated system^32^. With the discontinuous charge caused by the frustration in the film, an interfacial dipole is formed. It produces electrostatic potential barrier, and influences the polarization state at layers^33, 34^. That can be regarded as the basic reason for the enhanced polarization. The frustrated system is the most common system of magnetic order varies with space, and it is approved that the ferroelectricity is the breaking of space inversion by magnetic order^35^. The space inversion symmetry of magnetic ordering is broken in the as-grown multilayer structure, and induces the ferroelectricity. The effect can be phenomenologically interpreted in terms of Lifshiz–Landau theory^16^, the electric polarization *P* induced by magnetization *M* can be written as

1 $$P\propto [(M\bullet \partial )M-M(\partial \,\bullet \,M)]$$

It shows that various magnetic order in a magnetic frustrated system can induce local electric polarization in the magnet. When the induced electric polarization does not cancel out, ferroelectricity takes place. Therefore, we accredit the frustration at the interface as the extremely critical part to the emergence of ferroelectricity.

In the temperature region of 40 to 300 K, the *P-E* hysteresis loops are suppressed, indicating the paraelectric property of the SL film. In the absence of external forces, charged particles play random thermal motion. With increasing the temperature, the thermal motion exacerbates, which perturbs the charge order and leads to the offset of free polarization resulting in the decrease of polarization.

**Figure 3:**
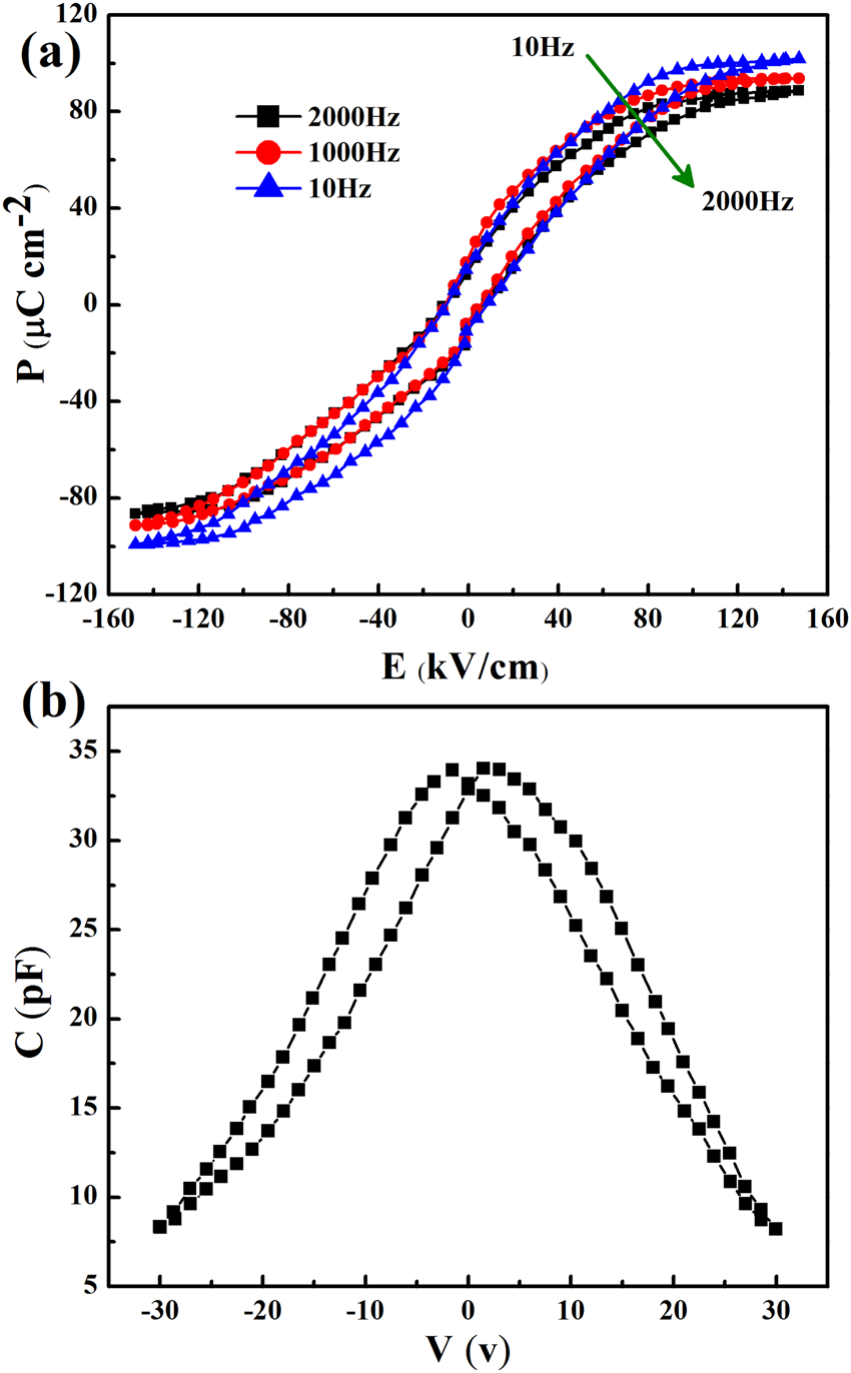
Evidence of ferroelectric behaviour in a SL with layer period of (3, 15). (**a**) *P-E* hysteresis loops measured at T = 30 K from 10 Hz to 2 kHz. (**b**) Capacitance–voltage (*C-V*) characteristic loop measured at T = 30 K.

Further, in order to identify and avoid artifacts in ferroelectric measurements, we have to circumvent space charges effects that have frequency-dependence and thermal excitation characteristics. Therefore, we test the samples by high-frequency at a low temperature. The *P-E* hysteresis loops are measured at different frequencies at 30 K, as shown in Fig. 3(a). It indicates the weak frequency dependence of the *P-E* hysteresis, which excludes contribution from extrinsic effects such as space charges, interface traps or charge leakage.

To further confirm that the sample is ferroelectric below 30 K, investigations are carried on the capacitance-voltage (*C-V*) characteristics of the SLs film under 2 kHz from 10 K to 300 K. Figure 3(b) displays representatively the *C-V* loop at 30 K. A butterfly loop behavior observed below 30 K suggests that the as-grown film exhibits ferroelectric property. Above 40 K, the *C-V* loops are also suppressed, which is consistent with the *P-E* loops. A *C-V* loop will appear the characteristic butterfly shape only when the material is ferroelectric^36^. Furthermore, the butterfly-loop has a strong symmetry and double peaks, which can rule out the space charge effect^37^. In addition, the applied electric fields corresponding to two maximum capacitances are close to the coercive field observed in Fig. 2(b).

**Figure 4:**
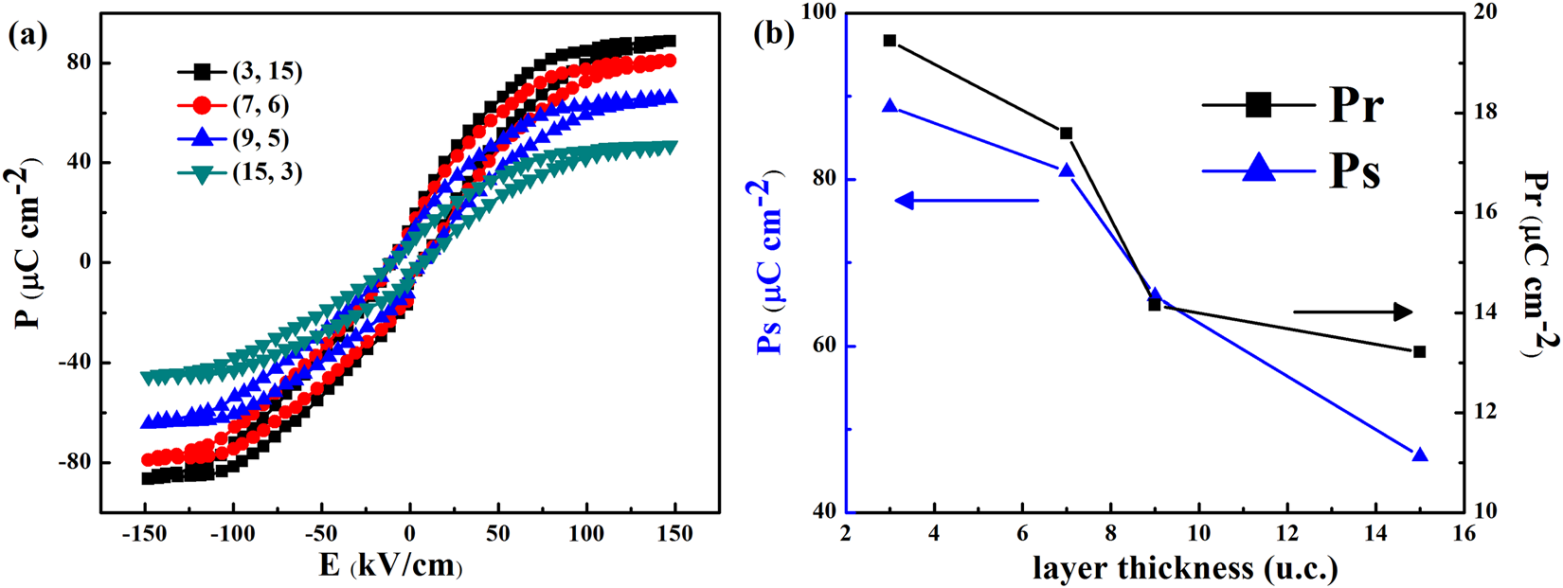
Evolution of ferroelectricity with layer periodicity. (**a**) *P-E* hysteresis loops for (3, 15), (7, 6), (5, 9) and (15, 3) SLs measured at 30 K at the frequency of 2 kHz. (**b**) Comparison of the thickness dependence of *P*_*s*_ *and P*_*r*_ for various SLs at T = 30 K. *Pr* and *Ps* represent the remnant polarization and the saturation polarization, respectively.

Moreover, in order to investigate the effect of periodicity on the polarization, SL films are prepared with fixed total thickness of 51.25 nm and different (*n, m*) = (3, 15), (7, 6), (9, 5) and (15, 3). The *P-E* hysteresis loops of SLs with different periodicities *m* or different sublayers thickness *n* are also investigated from 10 K to 300 K. As a representative, the *P-E* hysteresis loops at 30 K are shown in Fig. 4(a). Figure 4(b) illustrates *P*_*r*_ and *P*_*s*_ as a function of the thickness *n*, respectively. With decreasing the thickness of the sublayers *n*, the enhanced polarizations are observed. It reveals a strong thickness-dependence of the electric properties of the SLs. It is therefore, apparent that the interfaces play a central role in the emergent ferroelectric properties. The periodicity of the SLs affects the magnitude of the polarization. In the tricolor structure, the polarization induced by frustration at the interfaces cannot be offset. Therefore, the toroidal polarization will be accumulated with increasing the interfaces^24^.

**Figure 5:**
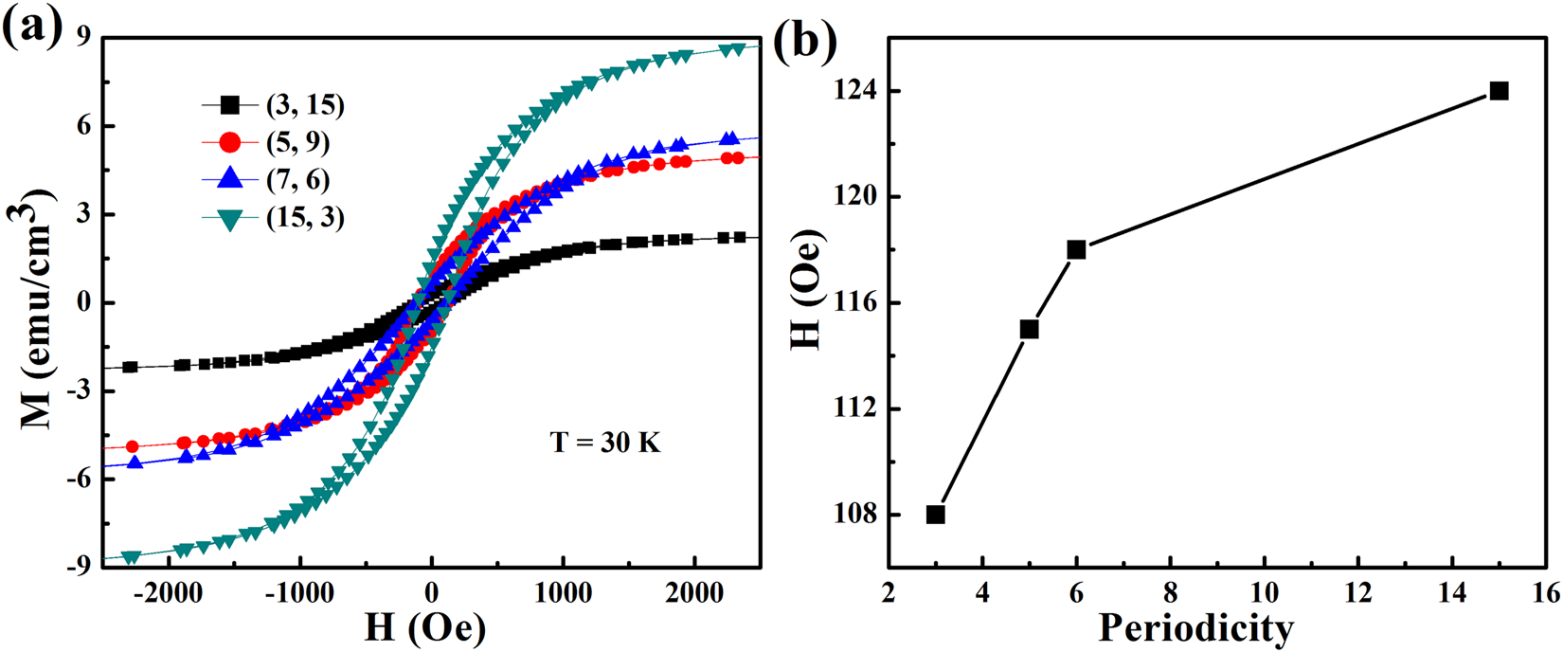
Magnetic measurements. (**a**) *M-H* hysteresis loops for (3, 15), (7, 6), (5, 9) and (15, 3) SLs measured at 30 K. (**b**) Comparison of the thickness dependence of magnetic coercivity for various SLs at T = 30 K.

It is necessary to study the magnetic properties of the film, so we logically measure the magnetization hysteresis (*M-H*) loops of the SLs with different periods at 30 K, seen from Fig. 5(a). The samples with *m* = 3, 5, 6 and 15 periods have the magnetic coercivity of 108, 115, 118 and 124 Oe, as illustrated in Fig. 5(b). All samples appear obvious ferromagnetism. The ferromagnetism can be attributed to the exchange interaction of Mn^3+^ and Mn^4+^. In the tri-color structure, the presence of Mn^3+^/Mn^4+^ mixed valency in the interlayers or at the interface gives rise to exchange coupling which causes the appearance of magnetism in our samples. Moreover, we note that the magnetic coercivity presents definitely a strong periodic dependence. The magnetism of samples reduce with increasing the periodicity *m* or decreasing the thickness of sublayers *n*. In our opinions, the reason lies in the fact that the frustration in films enhances with increasing the periods of SLs.

In summary, we investigate the structural and ferroelectric properties of [(LSMO)_n_/(PCMO)_n_/(LSbMO)_n_]_m_ SLs films grown on Nb:SrTiO_3_ single crystal substrate by a laser molecular-beam epitaxy technology at 1053 K. We obtain high quality films with a remnant polarization *P*_*r*_ ~ 18.9 μC cm^−2^ and a saturation polarization *P*_*s*_ ~ 88.7 μC cm^−2^ at 30 K, respectively. We demonstrate the emergence of ferroelectricity in artificial SLs composed of materials that are non-ferroelectrics in their bulk form. The ferroelectric properties of the multilayer structures are enhanced with increasing SL periodicity *m*, which is attributed to the spatial variations of magnetizations induced by the frustration in multilayer films. Meanwhile, the frustration enhances with increasing SL periodicity m, and then the exchange interaction between Mn^3+^ and Mn^4+^ is weakened. This leads to the reduction of magnetic properties in superlattice films. Thus, the strong periodic dependence of magnetism of SLs presents the presence of frustration, and we contribute the frustration in the films as the extremely critical part to the presence of multiferroicity. Our results further verify the previous theoretical research of generating multiferroics experimentally paving a way for designing or developing the novel magnetoelectric devices based on manganite ferromagnets.

## Methods

Pr_0.9_Ca_0.1_MnO_3_, La_0.9_Sr_0.1_MnO_3_ and La_0.9_Sb_0.1_MnO_3_ targets are prepared by the standard solid state reaction technique using high-purity Pr_6_O_11_, CaCO_3_, MnO_2_, La_2_O_3_, SrCO_3_ and Sb_2_O_3_. The [(LSMO)_n_/(PCMO)_n_/(LSbMO)_n_]_m_ SLs are fabricated on the Nb:SrTiO_3_ (NSTO) single crystal substrate with the area of 5 × 10 mm^2^ using a laser molecular-beam epitaxy (L-MBE) deposition method. Thin films forming SLs are synthesized by repeating m times tri-layers consisting of n unit cells of LSMO, PCMO and LSbMO. The frequency of a KrF excimer laser with a wavelength of 248 nm is maintained at 1 Hz focused on LSMO, PCMO and LSbMO polycrystal targets. The substrate is kept at 1053 K, and an oxygen pressure of 0.5 Pa is maintained throughout the deposition process. The total thickness of about 51.25 nm estimated by a transmission electron microscope (TEM, JEOL 2100 F) is controlled through adjusting the deposition time. Aurum electrodes are sputtered on the surfaces of SL films to obtain Ohmic contact, and the size of Aurum electrodes are 1 × 1mm. In measurements, the SL films are placed in a Janis C300 closed circuit cryostat with quartz glass windows, and the polarization versus electric field (*P-E*) hysteresis loops are measured by the modified Sawyer-Tower circuit (Precision LC, Radiant) in the temperature range from 10 to 300 K. During the testing process, each temperature point is held for 10 min to allow enough thermal relaxation to reduce the experimental error. Furthermore, the magnetic hysteresis loops are measured by a Superconducting Quantum Interference Device (MPMS-XL-7).

## Electronic supplementary material

Supplementary Information
