# Supplementary material for: The Frustration-induced Ferroelectricity of a Manganite Tricolor Superlattice with Artificially Broken Symmetry
Source: Sci Rep. 2017 Jul 24;7:6201. doi: 10.1038/s41598-017-06640-y (PMC5524750; doi:10.1038/s41598-017-06640-y)
Supplement: Supplementary file 1 — Supplementary Information [file 41598_2017_6640_MOESM1_ESM.pdf]

The Frustration-induced Ferroelectricity of a Manganite Tricolor Superlattice with Artificially Broken Symmetry

Huanyu Pei[1], Shujin Guo[1], Lixia Ren[1], Changle Chen[1*], Bingcheng Luo[1], Xianglei Dong[1], Kexin Jin[1], Ren Ren[2] and Hafiz Muhammad Zeeshan[1]

[1]Shaanxi Key Laboratory of Condensed Matter Structures and Properties, Northwestern Polytechnical University, Xi'an, 710072, China
[2]Department of Physics, Xi'an Jiaotong University, Xi'an, 710072, China
[*] chenchl@nwpu.edu.cn

Supplementary Figure

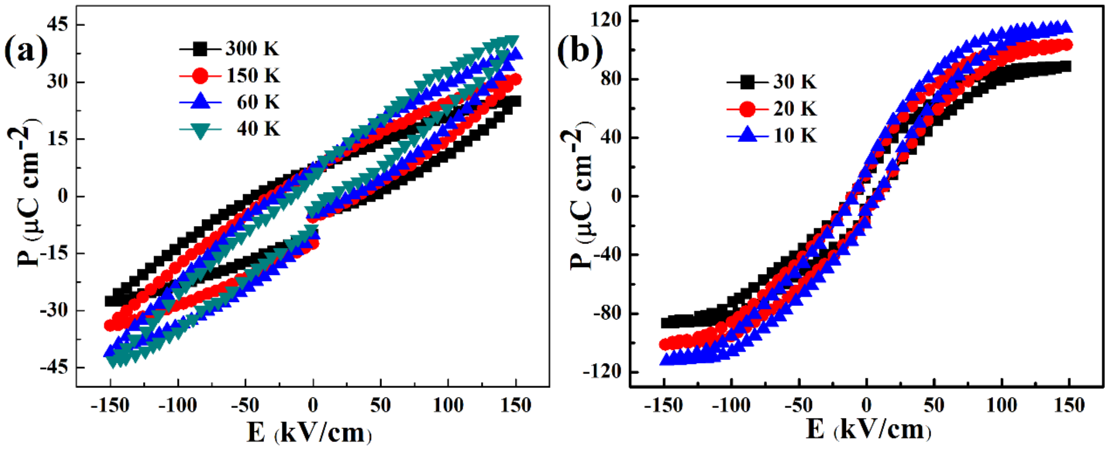

Supplementary Figure S1 The *P-E* loops above 40 K (a) and below 30K (b).
